# Changes in Oral Analgesic Prescribing in Japan between Fiscal Year 2014 and Fiscal Year 2022: A Nationwide Analysis Using Open Data from the National Database of Health Insurance Claims and Specific Health Checkups of Japan

**DOI:** 10.31662/jmaj.2026-0005

**Published:** 2026-05-29

**Authors:** Konosuke Tetsunaga, Tomonori Tetsunaga, Tomoko Tetsunaga, Yasumasa Kawada

**Affiliations:** 1 Concord College, Acton Burnell Hall, Acton Burnell, Shrewsbury, Shropshire, United Kingdom; 2 Department of Musculoskeletal Health Promotion, Faculty of Medicine, Dentistry, and Pharmaceutical Sciences, Okayama University, Okayama, Japan; 3 Department of Sports Medicine, Faculty of Medicine, Dentistry, and Pharmaceutical Sciences, Okayama University, Okayama, Japan; 4 Department of Cardiology, Japanese Red Cross Kochi Hospital, Kochi, Japan

**Keywords:** analgesics, prescription trends, neuropathic pain, nonsteroidal anti-inflammatory drugs, NSAIDs, acetaminophen

## Abstract

**Introduction:**

Nonsteroidal anti-inflammatory drugs (NSAIDs) have traditionally been central to pharmacological pain management in Japan, but population aging and increased use of mechanism-based analgesics may be changing prescribing patterns. We compared oral analgesic prescribing between fiscal year (FY) 2014 and FY2022 using nationwide claims-based open data, focusing on age and sex differences.

**Methods:**

We analyzed aggregated prescription volumes from the 1st and 9th editions of the National Database of Health Insurance Claims and Specific Health Checkups of Japan Open Data for FY2014 (April 2014-March 2015) and FY2022 (April 2022-March 2023). Analgesics were grouped as acetaminophen, NSAIDs, cyclooxygenase-2 selective inhibitors, weak opioids, duloxetine, neuropathic pain medications, and neurotropin. We calculated population-based prescription volume rates (per 1,000 population) using official population estimates and summarized patterns by sex and age group (<20, 20-64, ≥65 years). We also calculated within-age-group composition (percent share). Poisson regression with log(population) offset estimated incidence rate ratios (IRRs) comparing FY2022 versus FY2014 using robust standard errors; NSAID IRRs were further examined by age-sex strata.

**Results:**

Total prescription volume increased from 3.69 × 10^9^ in FY2014 to 6.09 × 10^9^ in FY2022. Rates per 1,000 population increased in all age groups, with the largest absolute increase among adults aged ≥65 years. NSAIDs remained the most frequently prescribed class by rate in adults aged 20-64 years, but composition shifted away from NSAIDs toward acetaminophen and neuropathic pain medications, most notably in older adults. Overall IRR was 1.55, with large increases for acetaminophen (2.51), neuropathic pain medications (2.14), duloxetine (2.29), and weak opioids (1.88), while NSAIDs changed modestly (1.15). NSAID rates decreased in older female strata (IRR <1 from ages 70-74 years).

**Conclusions:**

Oral analgesic prescribing in Japan increased substantially between FY2014 and FY2022, alongside age-specific shifts away from NSAIDs toward acetaminophen and neuropathic pain medications, particularly among older adults.

## Introduction

Pain management has traditionally relied on nonsteroidal anti-inflammatory drugs (NSAIDs) as the first-line treatment, particularly for musculoskeletal disorders in orthopedic practice. However, increasing recognition of distinct pain mechanisms―particularly neuropathic and chronic pain (pain persisting or recurring for >3 months)―has shifted attention toward alternative pharmacological strategies. The guidelines of several medical societies now recommend the use of medications specifically targeting neuropathic pain, such as gabapentinoids (pregabalin and mirogabalin), serotonin-norepinephrine reuptake inhibitors, tricyclic antidepressants, and weak opioids such as tramadol ^(1), (2)^.

In recent years, international studies have reported substantial changes in analgesic prescribing patterns. For example, the use of gabapentinoids has increased markedly in countries such as the United Kingdom, Denmark, and the United States ^(3), (4), (5)^, whereas trends in tramadol prescription have varied across countries, with both increases and decreases reported depending on national policies and clinical practice guidelines ^(6), (7)^. The use of traditional NSAIDs has either decreased or remained stable in many regions, partly due to concerns over their gastrointestinal, renal, and cardiovascular side effects ^(5), (8)^.

Despite these global trends, longitudinal evidence is limited regarding the evolution of analgesic prescribing practices in Japan. Earlier studies have indicated a continued dominance of NSAIDs in clinical settings, whereas more recent data suggest increased prescribing of pregabalin and weak opioids, particularly for chronic low back pain and osteoarthritis ^(9), (10)^. However, these trends have not been systematically evaluated across time periods via comprehensive prescription databases.

This study aimed to analyze trends in the prescription of analgesics in Japan via the National Database of Health Insurance Claims and Specific Health Checkups of Japan (NDB) Open Data, focusing on a comparison between the fiscal years (FYs) 2014 and 2022. Specifically, we examined changes in the use of NSAIDs, neuropathic pain medications, and weak opioids during this period. Understanding these trends is essential for aligning future prescribing practices with international standards and the specific needs of the Japanese patient population.

## Materials and Methods

### Data sources

We used publicly available aggregated data from the NDB Open Data published by the Ministry of Health, Labor, and Welfare. The NDB Open Data provides tabulated claims-based information, including prescription drug statistics stratified by sex and age group, derived from Japan’s nationwide health insurance claims database. We analyzed FY2014 (April 2014-March 2015) and FY2022 (April 2022-March 2023) ^(11), (12)^, corresponding to the 1st and 9th editions, respectively, of the NDB Open Data. These two editions were selected to provide an overview of long-term changes using the earliest and the most recent editions available at the time of analysis with comparable stratification by sex and 5-year age group. This two-time-point comparison summarizes the net change over the eight-year interval and does not assume a linear annual trend between FY2014 and FY2022.

Prescription data for oral analgesics were obtained from the “prescription drugs” tables stratified by sex and 5-year age groups. In the NDB Open Data, prescription volume is reported as “prescription quantity,” where the unit follows the drug master (e.g., tablet/capsule), and the quantity is calculated as the product of the daily dose (or per-dose amount) and the number of prescription days (or dosing occasions). Each strength/formulation is tabulated as a separate product; we aggregated product-level quantities within each pharmacological class without converting to dose equivalents (e.g., mg) or defined daily doses. Our analysis focused on oral formulations and did not include topical preparations such as NSAID patches.

Analgesics were categorized as acetaminophen-containing products, nonselective NSAIDs, cyclooxygenase-2 (COX-2) selective inhibitors (celecoxib), weak opioids (tramadol and tramadol/acetaminophen combinations), duloxetine, neuropathic pain medications (gabapentinoids: pregabalin and mirogabalin), and neurotropin (a nonprotein extract used in Japan for pain-related indications).

The NDB Open Data prescription drug tables list only top-ranked products within each therapeutic category and apply masking to small cells according to the publication criteria. For example, the 1st edition presents the top 30 products per category, whereas the 9th edition applies a broader upper-rank restriction (e.g., top 100/300/500 depending on category). Therefore, class-level totals in this study represent the sum of the published top-listed products and may not fully capture all prescriptions; differences in listing thresholds and masking specifications across editions may affect the comparability of absolute totals between FY2014 and FY2022. Only aggregated data were used; no individual-level or identifiable information was included. Therefore, ethics approval and patient consent were not required.

To compute population-based rates (per 1,000 population), population denominators by sex and 5-year age groups were obtained from the official Population Estimates published by the Statistics Bureau of Japan (e-Stat) and aggregated as needed for presentation ^(13)^.

### Statistical analysis

Prescription volume data and drug-class definitions were as described in the “Data sources” section. Throughout the manuscript, “prescription volume” refers to aggregated prescription quantities rather than unique patients; therefore, population-based rates represent prescription volume per population and should not be interpreted as treatment prevalence. Descriptive analyses were conducted to compare prescription volumes between FY2014 and FY2022. Total prescription volumes by drug class were tabulated, and overall composition (percent share) was calculated as follows: (drug-class volume / total analgesic volume) × 100. Sex-specific prescription volumes by drug class were presented for each fiscal year.

We calculated population-based prescription volume rates (prescription volume per 1,000 population) within each age group by dividing prescription volumes by the corresponding population denominators and multiplying by 1,000. Population denominators by sex and 5-year age groups were obtained from official Population Estimates (as of October 1 of each year) and aggregated into three age categories (<20, 20-64, and ≥65 years) for presentation to represent prescription volume per population rather than proportion of patients treated. We calculated within-age-group composition (percent share) for each drug class as follows: (drug-class volume within an age group / total analgesic volume within the same age group) × 100. This was done to display within-group distribution (proportion/share) rather than population-based rates.

To quantify changes in prescription volume rates between FY2014 and FY2022 while accounting for population denominators, we fitted Poisson regression models with a log link and the log of the population as an offset. Age-adjusted models included fiscal year and 5-year age group as covariates. Exponentiated coefficients were interpreted as incidence rate ratios (IRRs) comparing FY2022 with FY2014. Robust (sandwich) standard errors were used to account for potential overdispersion. We also fitted sex-adjusted Poisson regression models including fiscal year and sex as covariates, with log(population) used as an offset, to estimate sex-adjusted IRRs for each drug class (Supplementary Table S1). For NSAIDs, we also estimated age-sex stratum-specific IRRs by fitting Poisson models separately within each 5-year age group and sex stratum, using log(population) as an offset (Supplementary Table S2 and Supplementary Figure S1). For NSAIDs, we evaluated effect modification by including a three-way interaction term between fiscal year, sex, and an indicator of older age (≥70 years); the estimated interaction term (year×female×age≥70) is reported in Supplementary Table S3. Where appropriate, we also report an annualized IRR, calculated as exp{ln(IRR) / 8}, to express the average multiplicative change per year over the 8-year interval. Although the data represent nationwide aggregated counts, we used Poisson models to quantify rate ratios and provide interval estimates under a count-process model. All analyses were performed using SPSS version 29.0 (IBM Corp).

## Results

### Overall prescription volume and overall composition

Before describing prescribing changes, we note that the total population decreased from 127.1 million in FY2014 to 124.9 million in FY2022, while the proportion aged ≥65 years increased from 26.0% to 29.0% based on the population denominators used for rate calculations. All results are based on aggregated prescription quantities and do not represent unique patients.

**Figure 1.**
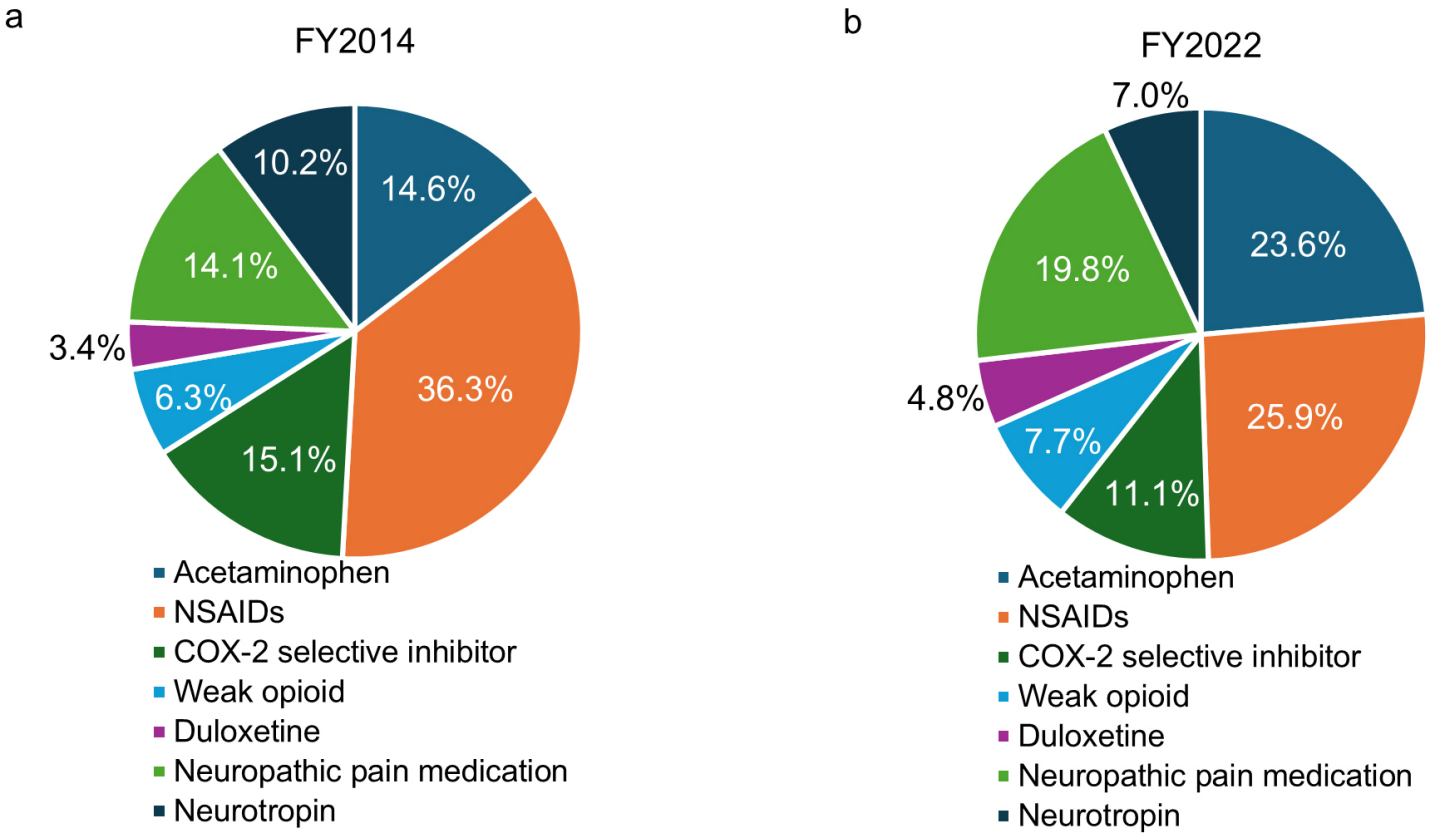
Proportions (percent share) of analgesic prescription volume. Charts show the proportions of each analgesic class for FY2014 (a) and FY2022 (b). The proportion of NSAIDs decreased from FY2014 to FY2022, whereas the proportions of acetaminophen and neuropathic pain medications increased. COX-2: cyclooxygenase-2; FY: fiscal year; NSAID: nonsteroidal anti-inflammatory drug.

The total prescription volume of oral analgesics increased from 3.69 × 10^9^ prescription quantity units (as defined in the NDB Open Data) in FY2014 to 6.09 × 10^9^ prescription quantity units in FY2022 (Table 1). In FY2014, NSAIDs accounted for the largest share of prescription volume (36.3%), whereas in FY2022, the proportion of NSAIDs decreased to 25.9% with concurrent increases in acetaminophen (14.6% to 23.6%) and neuropathic pain medications (14.1% to 19.8%) (Figure 1).

**Table 1.** Total Prescription Volume by Analgesic Class.

Drug class	FY2014	FY2022
Acetaminophen	538,937,065	1,436,095,283
NSAIDs	1,337,759,454	1,579,308,019
COX-2 selective inhibitor	557,954,017	677,226,826
Weak opioid	231,997,338	471,695,603
Duloxetine	124,200,324	293,700,413
Neuropathic pain medication	520,668,393	1,209,097,456
Neurotropin	376,932,756	425,951,026
Total	3,688,449,349	6,093,074,628

COX-2: cyclooxygenase-2; FY: fiscal year; NSAID: nonsteroidal anti-inflammatory drug.

### Sex-specific distribution

**Figure 2.**
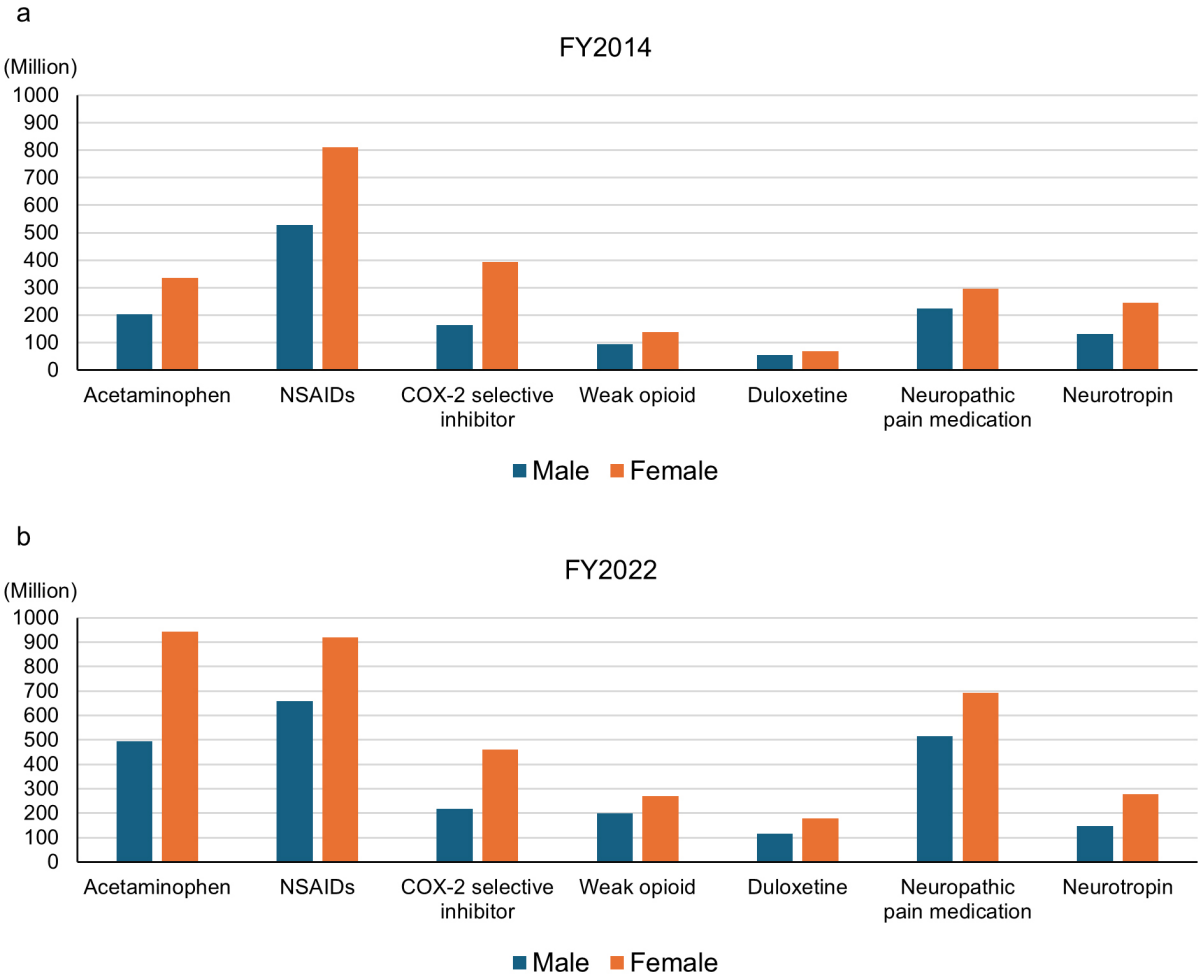
Prescription volume of oral analgesics by sex (million prescription quantity units), FY2014 and FY2022. The bar graphs show the prescription volume for FY2014 (a) and FY2022 (b). In both years, the total prescription volume was greater in females than in males. In females, acetaminophen was the most frequently prescribed analgesic in FY2022. COX-2: cyclooxygenase-2; FY: fiscal year; NSAID: nonsteroidal anti-inflammatory drug.

Across both fiscal years, females had higher prescription volumes than males for all analgesic classes (Figure 2). In FY2022, acetaminophen became the most frequently prescribed analgesic class among females, whereas NSAIDs remained the most frequently prescribed class among males (Figure 2).

### Age-specific population-based prescription volume rates

**Figure 3.**
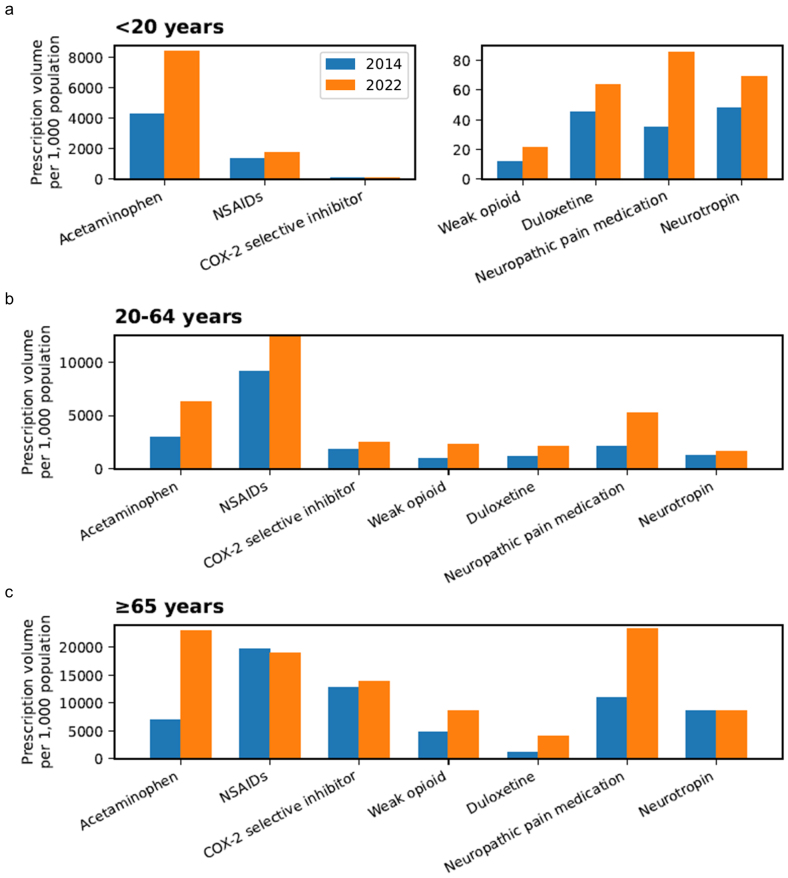
Population-based analgesic prescription volume rates by age group, FY2014 and FY2022. Bars show prescription volume rates per 1,000 population for each oral analgesic class in FY2014. Rates were calculated as follows: (prescription volume [aggregated prescription quantity] / corresponding population) × 1,000. Panels represent age categories: (a) <20 years, (b) 20-64 years, and (c) ≥65 years. In panel (a), the “<20 years” category is shown in two subpanels with different y-axis scales to improve visibility of less frequent classes. Prescription volume rates reflect volume per population and do not represent the proportion of patients treated. COX-2: cyclooxygenase-2; FY: fiscal year; NSAID: nonsteroidal anti-inflammatory drug.

Figure 3 shows population-based prescription volume rates (prescription volume per 1,000 population) by age group. The total prescription volume rate increased in all age groups from FY2014 to FY2022, rising from 5,866.6 to 10,611.1 per 1,000 population in those aged <20 years, from 19,570.6 to 32,588.1 in those aged 20-64 years, and from 65,214.6 to 100,500.2 in those aged ≥65 years (Figure 3).

In patients aged <20 years, acetaminophen was the predominant class and increased markedly (4,283.1 to 8,454.7 per 1,000 population), while NSAIDs increased modestly (1,350.4 to 1,786.1 per 1,000 population) (Figure 3a). In patients aged 20-64 years, NSAIDs showed the highest rates in both years (9,159.2 to 12,426.0 per 1,000 population) (Figure 3b). In patients aged ≥65 years, the largest absolute increases in rates were observed for acetaminophen (7,018.6 to 22,995.8 per 1,000 population) and neuropathic pain medications (11,084.3 to 23,410.7 per 1,000 population) (Figure 3c).

### Age-specific within-group composition (percent share)

**Figure 4.**
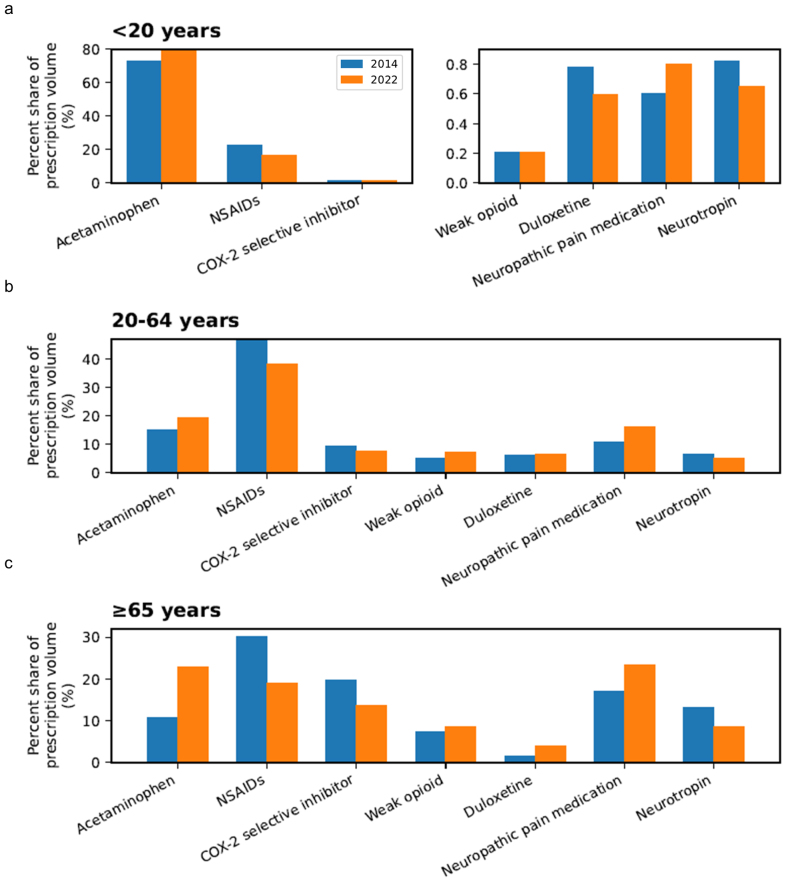
Age-group composition of analgesic prescription volume by drug class, FY2014 and FY2022. Bars show the within-age-group composition (percent share) of total oral analgesic prescription volume accounted for by each drug class in FY2014 and FY2022. Percent share was calculated as follows: (prescription volume of each drug class within an age group / total analgesic prescription volume within the same age group) × 100. Panels indicate (a) <20 years, (b) 20-64 years, and (c) ≥65 years. In panel (a), the “<20 years” category is shown in two subpanels with different y-axis scales to improve visibility of categories with small percent shares. These values represent within-group composition and are not population-based rates. COX-2: cyclooxygenase-2; FY: fiscal year; NSAID: nonsteroidal anti-inflammatory drug.

Figure 4 presents the within-age-group composition (percent share) of analgesic classes. Among patients aged <20 years, acetaminophen accounted for most prescription volume in both years (73.0% to 79.7%), whereas the share of NSAIDs decreased (23.0% to 16.8%) (Figure 4a). In patients aged 20-64 years, NSAIDs remained the largest class, but their share decreased from 46.8% to 38.1%, alongside increases in acetaminophen (15.1% to 19.4%) and neuropathic pain medications (11.0% to 16.0%) (Figure 4b). In patients aged ≥65 years, the composition shifted substantially, with neuropathic pain medications (17.0% to 23.3%) and acetaminophen (10.8% to 22.9%) becoming the two predominant classes by FY2022, while the share of NSAIDs decreased from 30.2% to 18.9% (Figure 4c).

### Poisson regression (IRR) and effect modification

In Poisson regression models with a log(population) offset, age-adjusted IRRs comparing FY2022 with FY2014 showed large increases in acetaminophen, neuropathic pain medications, duloxetine, and weak opioids, whereas changes in NSAIDs and neurotropin were comparatively small (Table 2).

**Table 2.** Age-Adjusted IRRs (FY2022 vs. FY2014) for Each Analgesic Class.

Drug class	IRR (FY2022 vs. FY2014)	95% CI	Annualized IRR (per year)	95% CI
Total	1.550	1.458-1.648	1.056	1.048-1.064
Acetaminophen	2.509	2.279-2.763	1.122	1.108-1.135
NSAIDs	1.147	1.055-1.248	1.017	1.007-1.028
COX-2 selective inhibitor	1.094	1.011-1.184	1.011	1.001-1.021
Weak opioid	1.875	1.685-2.086	1.082	1.067-1.096
Duloxetine	2.290	1.920-2.731	1.109	1.085-1.134
Neuropathic pain medication	2.141	1.982-2.312	1.100	1.089-1.110
Neurotropin	1.019	0.944-1.101	1.002	0.993-1.012

CI: confidence interval; COX-2: cyclooxygenase-2; FY: fiscal year; IRR: incidence rate ratio; NSAID: nonsteroidal anti-inflammatory drug.

In analyses stratified by age and sex, NSAID prescription volume rate ratios (FY2022 vs. FY2014) were <1 in older female strata (from ages 70-74 years onward), whereas decreases in males were observed mainly at older ages, suggesting age- and sex-related heterogeneity in temporal changes (Supplementary Table S2 and Supplementary Figure S1). Sex-adjusted Poisson models summarizing overall changes by drug class (Supplementary Table S1) showed increased prescription volume rates from FY2014 to FY2022 even after accounting for sex. In the interaction model for NSAIDs, the year×female×age≥70 term was <1 (IRR 0.937; 95% confidence interval 0.914-0.960; p < 0.001), indicating attenuated temporal change among females aged ≥70 years (Supplementary Table S3).

## Discussion

In this nationwide comparison using the NDB Open Data, we assessed changes in oral analgesic prescribing in Japan between FY2014 and FY2022 using prescription volumes, population-based prescription volume rates (per 1,000 population), and Poisson regression-based IRRs. Total prescription volume and population-based rates increased across all age groups, with the greatest absolute increase among adults aged ≥65 years. Although NSAIDs remained the largest class―particularly in non-elderly adults―the within-age-group composition shifted away from NSAIDs toward acetaminophen and neuropathic pain medications, most prominently among older adults. Age-adjusted IRRs supported these descriptive findings, indicating pronounced increases for acetaminophen and neuropathic pain medications and comparatively smaller changes for NSAIDs and neurotropin.

Sex-related differences in prescribing were evident in both fiscal years. Females consistently had higher prescription volumes than males across analgesic classes, and in FY2022, acetaminophen became the most frequently prescribed class among females, whereas NSAIDs remained the most frequently prescribed class among males (Figure 2). Sex-adjusted Poisson models (Supplementary Table S1) also indicated increased prescription volume rates between FY2014 and FY2022 across major drug classes after accounting for sex. First, sex-related differences in pain sensitivity have been attributed to hormonal influences, with women exhibiting a higher prevalence of chronic pain disorders and relatively weaker endogenous analgesic mechanisms ^(14), (15), (16), (17)^. Second, pain-related behaviors differ between men and women. Women are more likely to seek medical attention for pain at an early stage, whereas men are more likely to tolerate symptoms without consultation ^(18), (19), (20)^. This difference likely contributes to higher prescription rates among women. In addition, the prevalence of chronic pain is greater in women than in men, and the prescription volume is influenced by conditions that are more common in women, such as rheumatoid arthritis ^(21)^, osteoarthritis ^(22), (23)^, migraine ^(24)^, and fibromyalgia ^(25)^. Sex-specific pain conditions, including dysmenorrhea, may further contribute to higher analgesic demand among females ^(26), (27)^. Overall, the demand for analgesics tends to be greater among women ^(28), (29)^. These potential explanations are hypothesis-generating; because our analysis used aggregated prescription quantities without clinical indications or patient-level linkage, we could not directly evaluate the relative contribution of biological mechanisms, healthcare-seeking behavior, or prescribing preferences. Future patient-level studies linking prescriptions with diagnoses, indications, dose/duration, and outcomes are warranted.

Age-related differences were also clear when population denominators were incorporated. Population-based prescription volume rates increased from FY2014 to FY2022 in all age categories, with the highest rates and largest absolute increases in older adults (Figure 3). The prescribing mix evolved differently by age (Figure 4): acetaminophen dominated in individuals aged <20 years and increased further in share; NSAIDs remained the largest class in adults aged 20-64 years but declined in share as acetaminophen and neuropathic pain medications increased; and in adults aged ≥65 years, acetaminophen and neuropathic pain medications became the two predominant classes by FY2022, while the share of NSAIDs declined markedly. These patterns were consistent with age-adjusted IRRs (Table 2), which showed large relative increases for acetaminophen, neuropathic pain medications, duloxetine, and weak opioids compared with more modest changes for NSAIDs and COX-2 selective inhibitors and relatively small changes for neurotropin after age adjustment.

NSAIDs remained central to analgesic prescribing, particularly in non-elderly adults, consistent with their effectiveness for acute inflammatory and musculoskeletal pain. However, the declining proportional share of NSAIDs alongside increased use of non-NSAID options suggests a gradual diversification of pharmacologic strategies. In older adults, stratified analyses indicated heterogeneity by sex and age: NSAID IRRs decreased in older female strata (IRR <1 from the 70- to 74-year stratum), with decreases also observed in older male strata (Supplementary Table S2 and Supplementary Figure S1). This pattern is clinically plausible given the age-dependent risk profile of NSAIDs and the resulting tendency toward more cautious NSAID use in older adults. Consistent with these stratified estimates, the three-way interaction term (year×female×age≥70 years) was <1 (IRR 0.937), indicating additional attenuation of temporal change among older females.

The most prominent class-specific increase was observed for acetaminophen, with particularly large absolute increases among older adults (Figure 3). This increase may partly reflect time-varying contextual factors during the COVID-19 period. During the pandemic, acetaminophen was widely used for symptomatic relief of fever and viral symptoms, and early uncertainty regarding NSAIDs in COVID-19 may have reinforced a preference for acetaminophen in some settings ^(30), (31)^. In addition, pandemic-related changes in healthcare utilization and prescribing practices (e.g., consultation patterns and broader use of antipyretic/analgesic therapy outside conventional pain-management contexts) could have contributed to higher prescription quantities in FY2022. However, because this study is based on aggregated prescription quantities without indications and compares only two fiscal years, causal attribution to COVID-19-related factors is not possible. Acetaminophen may also have been selected more frequently as an alternative to NSAIDs in older adults; therefore, the increase in FY2022 likely reflects both analgesic prescribing practice and broader public health context.

In parallel, the increasing shares and IRRs for neuropathic pain medications and duloxetine suggest greater recognition and pharmacologic treatment of neuropathic and chronic pain, particularly in older adults (Figure 3 and 4; Table 2). This pattern aligns with the broader shift toward mechanism-based and multimodal approaches for chronic pain, in which gabapentinoids and serotonin-norepinephrine reuptake inhibitors are commonly used options, and weak opioids such as tramadol may be employed in selected situations ^(1), (2)^. Increased prescribing of these agents may also reflect a growing burden of chronic painful conditions with neuropathic components and a need for alternatives when NSAIDs are less suitable. However, these mechanistic interpretations are offered as contextual discussion rather than indication-specific conclusions because indications and pain duration/type are not available in the aggregated NDB Open Data.

Importantly, the observed shift away from NSAIDs should not be interpreted as uniformly “safer” because alternative analgesics have distinct risks. Acetaminophen can cause severe hepatotoxicity, particularly in the setting of overdose or unintentional cumulative exposure ^(32)^. Duloxetine carries warnings regarding suicidal thoughts and behaviors in younger populations and requires appropriate monitoring ^(33)^. Gabapentinoids are associated with central nervous system (CNS) adverse effects and have warnings regarding serious breathing problems in patients with respiratory risk factors or those receiving concomitant CNS depressants ^(34), (35)^. Tramadol can be associated with seizures and serotonin syndrome, particularly with interacting medications ^(36), (37)^. Accordingly, the evolving prescribing mix observed in FY2022 likely reflects a move toward risk-stratified and mechanism-based selection rather than a simple transition to uniformly lower-risk therapy.

Our findings are broadly consistent with international reports demonstrating similar shifts in analgesic use. Kurdi et al. ^(3)^ reported a 205%-207% increase in gabapentinoid prescriptions in the United Kingdom over one decade, reflecting an increased emphasis on neuropathic pain treatment in primary care. Likewise, Pottegård et al. ^(4)^ reported a nearly fourfold increase in gabapentinoid use in Denmark between 2010 and 2023, underscoring similar movements across diverse healthcare systems. In the United States, Gorfinkel et al. ^(5)^ reported that nonopioid prescriptions, including gabapentinoids and acetaminophen, rose significantly between 2014 and 2018. Comparative European studies also reinforce this trend. Bedene et al. ^(8)^ reported that countries such as the Netherlands have shifted toward non-NSAID options in aging populations, particularly for chronic pain, as part of safer prescribing initiatives. In contrast, trends in tramadol use vary across countries: Kim et al. ^(6)^ noted a dramatic increase in tramadol use in Korea, whereas Rasmussen et al. ^(7)^ reported a declining trend in Denmark, reflecting local regulatory and cultural factors. Taken together, these studies support the interpretation that Japan’s prescribing changes align with global movements toward multimodal pain management while also being shaped by domestic demographic and public health dynamics.

This study has several limitations. First, we used aggregated NDB Open Data without diagnoses or individual-level linkage; therefore, we could not confirm indications or appropriateness (e.g., acetaminophen may be prescribed for fever and duloxetine for depression) or adjust for patient-level factors such as comorbidities, dose, duration, concomitant medications, or outcomes. Second, prescription volume reflects “prescription quantity” in drug-master standard units and does not represent unique patients or standardized doses; moreover, our analysis included oral formulations only and did not capture topical NSAID preparations. Third, the NDB Open Data prescription tables list only top-ranked products and mask small cells, and listing thresholds differ across editions, which may affect the comparability of absolute totals. Finally, because only two fiscal years were compared, the timing of changes cannot be identified, and time-varying factors (including the COVID-19 pandemic) may have influenced prescribing patterns.

In conclusion, from FY2014 to FY2022, oral analgesic prescribing in Japan increased substantially in both total volume and population-based rates across all age groups. Over the same period, within-age-group prescribing shifted away from NSAIDs toward acetaminophen and neuropathic pain medications, most markedly among older adults; Poisson models with log(population) offsets showed higher age-adjusted rates in FY2022, with the largest relative increases for acetaminophen and neuropathic pain medications. Females consistently had higher prescription volumes than males, and NSAID rate changes in older adults differed by age and sex. These findings describe oral prescriptions captured in the NDB Open Data and do not include topical NSAID preparations such as patches.

## Article Information

### Acknowledgments

We would like to thank FORTE (www.forte-science.co.jp) for English language editing.

### Author Contributions

Konosuke Tetsunaga was responsible for study conception and design, acquisition and curation of the publicly available datasets, analysis and interpretation of the data, and drafting of the manuscript. Tomonori Tetsunaga contributed to the study concept and interpretation of the results and critically reviewed and revised the manuscript for important intellectual content. Tomoko Tetsunaga and Yasumasa Kawada contributed to the statistical analyses and interpretation of the findings. All authors read and approved the final manuscript.

### Conflicts of Interest

None

### Institutional Review Board Approval Code and Name of the Institution

This study was approved by the ethics committee of Okayama University Hospital (approval number K2511-032).

## Supplement

Supplementary Material Supplementary Figure S1. Stratum-specific IRRs of NSAID volume rates in FY2022 relative to FY2014, stratified by sex and age group.
The dashed horizontal line indicates IRR = 1.0 (no change); values >1 denote higher and values <1 denote lower NSAID volume rates in FY2022 compared with FY2014.
FY: fiscal year; IRR: incidence rate ratio; NSAID: nonsteroidal anti-inflammatory drug.
