# Supplementary material for: Changes in Oral Analgesic Prescribing in Japan between Fiscal Year 2014 and Fiscal Year 2022: A Nationwide Analysis Using Open Data from the National Database of Health Insurance Claims and Specific Health Checkups of Japan
Source: JMA J. 2026 May 29;9(4):942–51. doi: 10.31662/jmaj.2026-0005 (PMC13453779; doi:10.31662/jmaj.2026-0005)
Supplement: Supplementary file 1 [file 2433-3298-9-4-0942-s001.pdf]

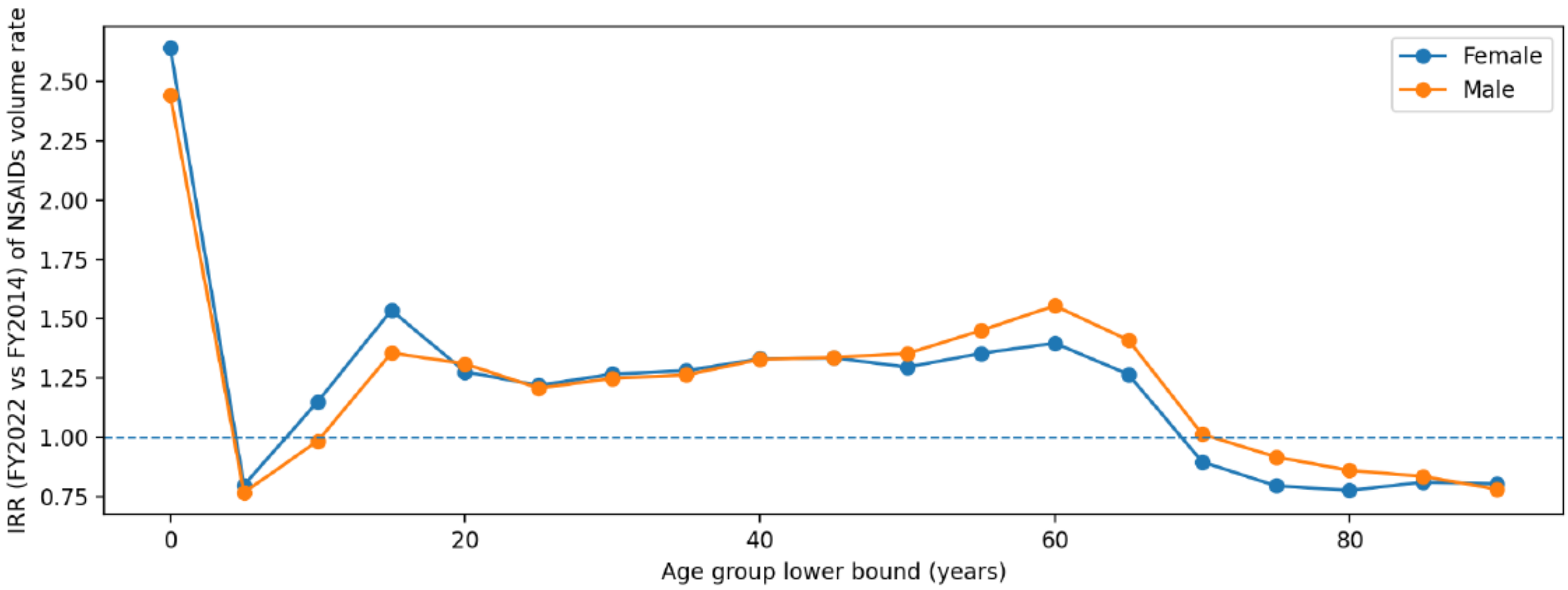

IRR (FY2022 vs FY2014) of NSAIDs volume rate
Age group lower bound (years)
Female
Male
2.50
2.25
2.00
1.75
1.50
1.25
1.00
0.75
0
20
40
60
80

Supplementary Table S1. Stratum-specific incidence rate ratios (IRRs) for NSAIDs prescription volume rates (FY2022 vs FY2014) by sex and 5-year age group.

Age group (years)	Female IRR (95% CI)	Male IRR (95% CI)
0–4	2.642 (2.587–2.699)	2.442 (2.366–2.521)
5–9	0.798 (0.795–0.801)	0.767 (0.764–0.770)
10–14	1.150 (1.148–1.151)	0.984 (0.982–0.985)
15–19	1.535 (1.534–1.537)	1.355 (1.354–1.357)
20–24	1.276 (1.275–1.277)	1.309 (1.308–1.310)
25–29	1.219 (1.218–1.220)	1.207 (1.206–1.208)
30–34	1.267 (1.266–1.267)	1.248 (1.248–1.249)
35–39	1.280 (1.280–1.281)	1.262 (1.262–1.263)
40–44	1.330 (1.330–1.331)	1.328 (1.328–1.329)
45–49	1.334 (1.334–1.335)	1.337 (1.336–1.337)
50–54	1.295 (1.295–1.296)	1.353 (1.352–1.353)
55–59	1.353 (1.353–1.354)	1.451 (1.450–1.451)
60–64	1.397 (1.396–1.397)	1.554 (1.553–1.555)
65–69	1.265 (1.264–1.265)	1.410 (1.409–1.410)
70–74	0.896 (0.896–0.896)	1.013 (1.012–1.013)
75–79	0.795 (0.795–0.795)	0.916 (0.916–0.917)
80–84	0.776 (0.775–0.776)	0.860 (0.860–0.860)
85–89	0.810 (0.8099–0.8104)	0.835 (0.834–0.835)
90+	0.804 (0.803–0.804)	0.780 (0.780–0.781)

NSAIDs, non-steroidal anti-inflammatory drugs; CI, Confidence Interval. Rates are prescription volume per 1,000 population (denominator: official Population Estimates). 95% CIs were calculated using a Poisson approximation on the log(IRR). IRR definition: IRR compares FY2022 with FY2014 for NSAIDs prescription volume rates within each age–sex stratum. Rate definition: rate = (prescription volume)/(population) (offset = log population).
Note: counts represent prescription volume from aggregated NDB Open Data (not unique patients).

Supplementary Table S2. Sex-adjusted incidence rate ratios (IRRs) for analgesic prescription volume rates per 1,000 population, comparing FY2022 vs FY2014.

Drug class	IRR (FY2022 vs FY2014)	95%CI	Annualized IRR (/year)	95%CI
Total	1.680	1.659–1.701	1.067	1.065–1.069
Acetaminophen	2.710	2.519–2.916	1.133	1.122–1.143
NSAIDs	1.201	1.149–1.255	1.023	1.017–1.029
COX-2 selective inhibitor	1.234	1.171–1.301	1.027	1.020–1.033
Weak opioid	2.068	1.988–2.151	1.095	1.090–1.100
Duloxetine	2.405	2.150–2.690	1.116	1.100–1.132
Neuropathic pain medication	2.362	2.345–2.379	1.113	1.112–1.114
Neurotropin	1.149	1.148–1.150	1.018	1.017–1.018

FY, fiscal year; CI, Confidence Interval; NSAIDs, non-steroidal anti-inflammatory drugs; COX, cyclooxygenase

Supplementary Table S3. Three-way interaction term (Year×Female×Age≥70 years) from Poisson regression models.

Drug class	IRR (Year×Female×Age≥70)	95% CI		P value
		Lower	Upper	
NSAIDs	0.937	0.914	0.960	<0.001

IRR, incidence rate ratios; CI, Confidence Interval; NSAIDs, non-steroidal anti-inflammatory drugs. IRR represents the multiplicative interaction for the change from FY2014 (reference) to FY2022 among females aged ≥70 years relative to males and younger age strata. The model used a log link with log(population) as an offset and robust (sandwich) standard errors; covariates included fiscal year, sex, 5-year age group, fiscal year×sex, fiscal year×age group, and fiscal year×sex×age≥70. Some strata were excluded where structural zeros prevented model estimation.
